# P-2089. Transition of Lyme Serological Testing to the Modified Two Tiered-Testing Algorithm (MTTT): Observations from a Commercial Laboratory

**DOI:** 10.1093/ofid/ofae631.2245

**Published:** 2025-01-29

**Authors:** Laura Gillim, Ajay Grover, Charles M Walworth, David Alfego

**Affiliations:** Labcorp, Burlington, North Carolina; Labcorp, Burlington, North Carolina; Monogram Biosciences/LabCorp, Laguna Beach, California; Labcorp, Burlington, North Carolina

## Abstract

**Background:**

2019 FDA clearance of Lyme disease serologic assays with new indications for use in a modified two-tiered testing (MTTT) algorithm marked the first significant change in Lyme serologic testing in over 20 years. In 2022 Labcorp® fully transitioned Lyme serological testing from the standard two-tiered testing (STTT) algorithm to the MTTT algorithm.

**Methods:**

Retrospective analysis of results reported for Lyme serological testing March through October to correlate with warmer months consistent with tick exposure, comparing samples received in 2019 and 2021 assessed with the STTT algorithm and samples received in 2022 and 2023 with the MTTT algorithm. Algorithm interpretation was based on APHL guidance.

**Results:**

In 2019 and 2021, a combined total of 314,409 individuals were tested from March through October using the Lyme STTT algorithm while a combined total of 597,142 individuals were tested in 2022 and 2023 using MTTT during the same time frame. The populations of individuals tested by STTT and MTTT did not differ significantly in sex (STTT: 57.2% female, MTTT: 57.4%), age (STTT: mean 51 ± 29.0 years, MTTT: 51.0 ± 30.0 years) or geographic distribution (by U.S. census bureau region). An overall increase in positivity was observed with the MTTT (6.4%) compared to the STTT (3.2%) and was specifically observed in the percentage of individuals with results consistent with recent or remote past infection (tier 1+/tier 2 IgG+ or both IgG+ IgM+). A decrease in the percentage of results consistent with acute or recent infection (tier 1+/tier 2 IgM+) was observed with MTTT (1.1%) versus STTT (1.5%). 11.6% of Lyme MTTT orders included a concurrent order for Lyme immunoblot. MTTT and immunoblot results were discordant for 9.1% of samples. Of the discordant samples 43% had detectable IgG and/or IgM on the 2^nd^ tier MTTT but were negative by immunoblot.

**Conclusion:**

The transition from the STTT to MTTT algorithm revealed higher rates of serological positivity for Lyme disease. Analysis of samples tested by both MTTT and immunoblot indicates that MTTT assays exhibit greater sensitivity, aligning with findings in peer-reviewed literature. Some clinicians ordered both MTTT and immunoblot tests, suggesting a need for education on updated Lyme testing recommendations.

**Disclosures:**

Laura Gillim, PhD, Labcorp: Employee|Labcorp: Stocks/Bonds (Public Company) Charles M. Walworth, MD, Labcorp: Employee|Labcorp: Employee|Labcorp: Stocks/Bonds (Public Company) David Alfego, PhD, Labcorp: Employee|Labcorp: Stocks/Bonds (Public Company)

